# Cytomegalovirus infection and progressive differentiation of effector-memory T cells

**DOI:** 10.12688/f1000research.15753.1

**Published:** 2018-09-26

**Authors:** Iris N. Pardieck, Guillaume Beyrend, Anke Redeker, Ramon Arens

**Affiliations:** 1 Department of Immunohematology and Blood Transfusion, Leiden University Medical Center, Albinusdreef 2, 2333ZA, Leiden, The Netherlands

**Keywords:** Cytomegalovirus, memory CD8 T cell, memory inflation, T cell differentiation

## Abstract

Primary cytomegalovirus (CMV) infection leads to strong innate and adaptive immune responses against the virus, which prevents serious disease. However, CMV infection can cause serious morbidity and mortality in individuals who are immunocompromised. The adaptive immune response to CMV is characterized by large populations of effector-memory (EM) T cells that are maintained lifelong, a process termed memory inflation. Recent findings indicate that infection with CMV leads to continuous differentiation of CMV-specific EM-like T cells and that high-dose infection accelerates this progression. Whether measures that counteract CMV infection, such as anti-viral drugs, targeting of latently infected cells, adoptive transfer of CMV-specific T cells, and vaccination strategies, are able to impact the progressive differentiation of CMV-specific EM-like cells is discussed.

## Introduction

Human cytomegalovirus (HCMV) is a highly prevalent virus that establishes a state of persistent infection ^1^. Primary infection rarely causes severe disease in immunocompetent individuals. However, infection of immunocompromised individuals (for example, untreated HIV and transplant patients) or congenitally infected children ultimately can result in serious disease and mortality ^2^. CMV is also considered to play a role in immune senescence, although its role herein is controversial ^3, 4^.

During primary HCMV infection, there is a strong natural killer cell response, which is succeeded by the formation of humoral and cellular immunity ^5^. CMV immunity comprises neutralizing antibodies and the generation of CMV-specific CD4 ^+^ and CD8 ^+^ T cells recognizing an extensive range of viral proteins. On average, the T-cell response to CMV is exceptionally high. About 10% of the memory T-cell compartment in blood is CMV specific ^6^ and therefore HCMV is considered one of the most immunogenic pathogens for humans. However, the range of T-cell frequencies in the blood of infected individuals is quite variable, ranging from barely detectable to very high (even above 40%), and this variance is likely caused by differences in the infectious dose and host-intrinsic factors. Importantly, HCMV infection has been demonstrated to be a major driver of the variation in the immune system by systems-level analysis ^7^.

Despite robust primary immune responses leading to control of primary infection, the virus is never cleared. The establishment of latent infection and subsequent recurrent viral reactivation from latency are related to numerous sophisticated immune evasion strategies of the virus. For example, CMV-encoded genes impair major histocompatibility complex (MHC) class I and II-restricted antigen processing and presentation, which suppresses CD8 ^+^ and CD4 ^+^ T-cell recognition ^8, 9^. CMV also prevents the activation of T cells by down-modulating co-stimulatory ligands on infected antigen-presenting cells ^10, 11^.

Although latent infection suggests a silent state, it has become evident that changes in the phenotype of virus-reactive cells occur during the course of persistent infection and that these changes are related to factors such as the initial dose of viral inoculum and aging. Here, we discuss recent findings regarding the differentiation of CMV-specific T cells and interventions that counteract CMV-associated perturbations that may impact T-cell differentiation.

## Progressive differentiation of cytomegalovirus-specific effector-memory T cells

The T-cell response to CMV is exceptional because of the large numbers of functional effector-memory-like (EM-like) cells that are induced and maintained lifelong in blood and tissue. This phenomenon, termed memory inflation ^12– 14^, relates to the low-level persistence of the virus, as demonstrated by viral latency and intermittent viral reactivation. In healthy hosts, the infectious dose is a strong determinant of the degree of memory inflation that occurs ^15^. The circulating EM-like T cells that are induced upon CMV infection express markers such as KLRG1 and CD44, whereas expression of CD62L, CD127 (IL-7Rα), and the co-stimulatory molecules CD27 and CD28 is downregulated or lost ^16– 18^. In tissues, not only circulating EM-like CMV-specific T cells but also CMV-specific non-recirculating tissue-resident memory (TRM) T cells are present. These TRM T cells, considered a distinct memory population ^19^, are characterized by CD69 expression and, depending on the tissue, also express CD103 ^20^. CMV-specific memory T cells with a central-memory (CM)-like phenotype (CD62L ^+^, CD127 ^+^, CD27 ^+^, CD28 ^+^, KLRG1 ^−^, and IL-2 ^+^) also exist and are thought to dominantly contribute to population expansion upon re-challenge ^21^. Systemic control of CMV infection likely depends on the collective contribution of the circulating and non-circulating CMV-specific T cells.

**Figure 1.**
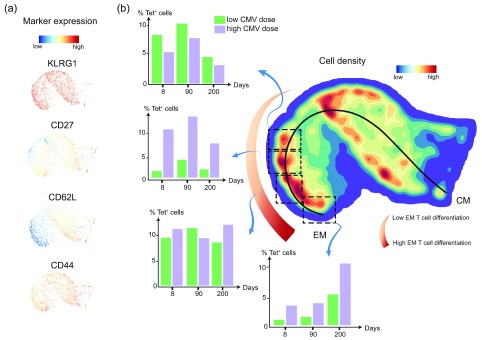
Progressive differentiation of cytomegalovirus (CMV)-specific effector-memory (EM) CD8 ^+^ T cells and its relation to the initial viral inoculum. C57BL/6 mice were infected intraperitoneally with a low (10 ^3^ plaque-forming units, or PFU) or a high (10 ^5^ PFU) dose of mouse CMV (MCMV)-Smith. Inflationary IE3-specific CD8 ^+^ T cells in blood were detected with major histocompatibility complex (MHC) class I tetramer (Tet) and stained for the cell surface markers CD62L, KLRG1, CD27, and CD44, which allow discrimination between central-memory (CM) and EM-like T cells at days 8, 90, and 200 after infection. ( **a**) IE3-specific cells were gated (Tet ^+^) and an equally down-sampled number of cells per sample was analyzed by Cytosplore. Single-marker expression of IE3-specific CD8 ^+^ T cells is shown as Approximated t-distributed Stochastic Neighbor Embedding (A-tSNE) scatterplots to visualize the intensity of the markers. ( **b**) A-tSNE plot depicts the pooled phenotypical data of the cell surface markers visualized as cell density clusters of the IE3-specific CD8 ^+^ T cells of low- and high-dose MCMV-infected mice for day 8, 90, and 200 time points after infection. In the A-tSNE plot, the differentiation path from the CM and EM phenotype is specified. The black curved line indicates the ongoing shift toward a higher advanced EM phenotype. Clusters showing different points in the differentiation path were selected and indicated by dashed line boxes. The percentage of IE3-specific CD8 ^+^ T cells in the selected clusters was determined and displayed in the corresponding bar graphs for each time point and viral inoculum.

With the use of novel computational tools that allow the analysis of cytometry data with much finer detail ^22, 23^, we recently discovered that CMV infection continuously affects the differentiation of the virus-specific EM-like cells ^24^. Inflationary T cells seem to undergo progressive differentiation unremittingly, and this was most clearly observed upon high-dose infection. Quantification of inflationary CMV-specific CD8 ^+^ T cells in different stages of EM differentiation performed with the previously described Cytosplore data set ^24^ revealed that the differentiation of these EM-like inflationary CMV-specific T cells strikingly increases over time ( Figure 1). High-dose infection accelerated this progressive profile of EM differentiation compared with a lower dose. This quantification unequivocally shows that CMV infection causes progressive EM T-cell differentiation that continues throughout the life span of the host and that the grade of infection (for example, low versus high) impacts the degree of circulating EM T-cell differentiation. It is currently unknown whether TRM T-cell differentiation is impacted by aging and infectious dose.

Progressive EM T-cell differentiation might be dependent on the differentiation of naïve and CM-like T cells into EM T cells ^17^ but as well on the stimulation and expansion of less EM-differentiated cells into more differentiated populations. CD27 expression, which is higher on less differentiated cells, gradually declines on the cell surface of inflationary EM T-cell populations over time. Thus, CD27 has a likely role in progressive EM T-cell differentiation and coincides with the requirement of CD27–CD70 interactions to support and maintain memory inflation ^25^. Moreover, CD27-expressing memory T cells can restore inflationary populations during latency ^26^. The anti-apoptotic molecule Bcl-2 accumulates over time and presumably is essential for the survival of the inflationary EM T cells ^27^. Correspondingly, the half-life of inflationary EM T cells is considerably longer than that of effector T cells, which have lower Bcl-2 levels ^28^. Together, these findings fuel the concept that less-differentiated EM-like T cells can accumulate into more differentiated EM cells and that these cells have a prolonged survival. Whether the cytokines IL-2 and IL-15, implicated in the maintenance of inflationary T cells ^28, 29^, also directly contribute to the differentiation of the T cells is unclear, but surely higher levels of CMV drive the T-cell differentiation to a more advanced EM phenotype.

Whether progressive EM T-cell differentiation is a non-functional response against the persistent presence of CMV antigens or a functional adaptation of the CMV-reactive T cells to keep control over CMV infection is unknown, but it may affect cellular function and senescence. In humans, latent CMV infection in older individuals resulted in lower protection rates after vaccination with an influenza vaccine ^30– 32^; however, neutral effects of CMV infection were also observed ^33– 36^. Possibly related to some of these studies is the finding that high-dose but not low-dose CMV infection impairs the development of a heterologous anti-viral T-cell response ^24, 37, 38^. In contrast to these possible negative outcomes of persistent CMV infection, recent studies are indicating positive effects. For example, it was shown that old mice infected with mouse CMV (MCMV) had a broader T-cell response compared with non-infected old mice after challenge with *Listeria monocytogenes* ^39^. Notably, positive effects of CMV infection early after infection have been well documented and may relate to a heightened innate immune activation status ^24, 35, 40^. Together, these studies imply that the progressive differentiation of CMV-specific EM-like T cells may be either negatively or positively affecting host immunity. CMV latency and its impact on T-cell responses thus may reflect a virus–host balance that can be impacted by the infectious dose and aging. Nevertheless, it is likely that lowering of the viral load is key to diminishing putative T-cell senescence, as lower viral loads lead to a reduction in EM T-cell differentiation ^15, 41^.

## Measures to counteract cytomegalovirus-associated perturbations and their impact on T-cell differentiation

In cases where CMV-associated perturbations are known to be a negative factor (for example, in congenital infection and viral reactivation after transplantation), measures to reduce the burden of CMV infection are being investigated. Several approaches, such as anti-viral drugs, treatments targeting latently infected cells, adoptive transfer of CMV-specific T cells, and (prophylactic) vaccines, have been developed.

Anti-viral drugs targeting CMV are commonly used for transplantation patients with clinical reactivation of CMV upon transplantation ^42^. In these patients, the use of anti-viral drugs can reduce viral load ^43^, but not much is known about the effect of anti-viral drugs on the differentiation of CMV-specific EM T cells. Whether anti-viral therapy can be used to reduce EM T-cell differentiation and improve heterologous immunity was recently experimentally assessed by Beswick *et al*. ^44^. Administration of valaciclovir to mice with an established MCMV infection resulted in a reduction of the magnitude of the MCMV-specific CD8 ^+^ T-cell response. This was accompanied by a less-differentiated phenotype of the residual CD8 ^+^ T cells compared with mice that received no anti-viral treatment. Treatment with valaciclovir also reduced influenza A viral loads upon challenge and reduced the differentiation of influenza-specific CD8 ^+^ T cells. However, CMV can adapt to become resistant to anti-viral treatment ^45^, suggesting that treatment with anti-viral drugs might not generally be effective in the long term.

A sophisticated way to target latent infection can be to manipulate the mechanisms used by CMV to avoid detection by the immune system ^46^. The viral protein UL138, expressed during latency, results in loss of multidrug resistance-associated protein-1 (MRP1) ^47^. The treatment of latently infected monocytes with vincristine, a cytotoxic agent normally exported by MRP1, resulted in specific ablation of these cells. Also, other genes involved in CMV latency (for example, US28 encoding a cell surface G-protein-coupled receptor ^48, 49^ and LUNA encoding a motif with deSUMOylase activity ^50^) could be targeted to clear CMV. Such treatments may be used to eliminate latently infected cells before transplantation. However, because a wide range of cells is latently infected during CMV infection, this therapy could result in unwanted side effects. But it is likely that a substantial reduction in latently infected cells, if effective, will diminish memory inflation and the ongoing EM T-cell differentiation.

Adoptive transfer of HCMV-specific T cells is a method used to restore CMV-specific immunity in transplant recipients and has been shown to reduce the risk for HCMV infection or reactivation or both ^51, 52^. For this type of treatment, it is expected that different subsets of CMV-specific T cells being transferred have different effects on protective immunity and viral load ^53^. Some studies have examined the relationship of the T-cell phenotype with the clinical outcome ^54– 56^. Positive correlations were found between population expansion and the number of CM-like cells within the transferred population. Weeks after transfer, the majority of the expanded CD8 ^+^ T cells nevertheless become highly differentiated. In-depth studies are required to assess the impact and level of progressive EM T-cell differentiation that occurs in adoptive T-cell transfer settings.

Prophylactic vaccination strategies have the potential to reduce the viral load of CMV. Several different CMV vaccination platforms have been developed ^57, 58^. Most of these concentrated on eliciting antibodies but some (also) aimed to induce robust CMV-specific T-cell immunity ^59– 61^. Vaccination with live-attenuated and replication-deficient CMV vectors seems to induce CD8 ^+^ T-cell responses undergoing less memory inflation, including the induction of the associated EM cell phenotype ^62, 63^. Vaccination with synthetic long peptides containing MCMV epitopes induces strong and polyfunctional CD8 ^+^ T-cell responses, but whereas responses to these epitopes are inflationary in the MCMV setting, they are non-inflationary in the peptide vaccine setting, which corresponds with their reduced EM-like phenotype ^64, 65^. Nevertheless, such vaccines are able to reduce viral load upon challenge with virulent CMV. It will be of interest to decipher the significance of the T-cell differentiation phenotype in relation to the effectiveness of CMV vaccines.

In conclusion, CMV infection results in a progressive differentiation of viral-specific EM T cells. The consequences of such a progressive differentiation may have both detrimental and beneficial effects on the virus–host balance and require further investigation. Such investigations may reveal opportunities to optimize immune function in CMV-seropositive people. Whether progressive differentiation is a distinctive property of the EM-like CMV-specific T cells undergoing inflation or whether progressive differentiation also occurs in other T-cell subsets (for example, TRM T cells) and in other infection settings remains to be explored.
